# Know when to run, know when to hide: can behavioral differences explain the divergent invasion success of two sympatric lizards?

**DOI:** 10.1002/ece3.22

**Published:** 2011-11

**Authors:** David G Chapple, Sarah M Simmonds, Bob BM Wong

**Affiliations:** School of Biological Sciences, Monash UniversityClayton, Victoria, Australia

**Keywords:** Exploratory behavior, introduction process, invasion ecology, *Lampropholis*, propagule pressure, social tendency

## Abstract

Invasive species represent a select subset of organisms that have successfully transitioned through each stage of the introduction process (transportation, establishment, and spread). Although there is a growing realization that behavior plays a critical role in invasion success, few studies have focused on the initial stages of introduction. We examined whether differences in the grouping tendencies and exploratory behavior of two sympatric lizard species could contribute to their divergent invasion success. While the nondirected activity of the two species did not differ, the invasive delicate skink (*Lampropholis delicata*) was found to be more exploratory than the congeneric noninvasive garden skink (*L. guichenoti*), which enabled it to more effectively locate novel environments and basking site resources. The delicate skink also exhibited a greater tendency to hide, which may act to enhance its probability of ensnarement in freight and cargo and decrease its likelihood of detection during transit. The grouping tendencies of the two species did not differ. Together, our results suggest that while the two species have an equivalent “opportunity” for unintentional human-assisted transportation, several pre-existing behavioral traits may enhance the success of the delicate skink in negotiating the initial stages of the introduction process, and subsequent post-establishment spread.

## Introduction

Invasive species do not represent a random subset of the world's biodiversity (Suarez and Tsutsui 2008). Each year, human activities result in the unintentional movement of individuals from thousands of different species to areas outside of their native range (Lockwood et al. 2007). While a select group of these introductions successfully transition through the gauntlet of transportation, establishment, and spread to ultimately become invasive, the vast majority fail to proceed through the entire introduction process (Suarez et al. 2005; Ward et al. 2006; Lockwood et al. 2007). Identifying the factors that underlie the ultimate fate of stowaways represents a central focus of invasion ecology (Kolar and Lodge 2001; Hayes and Barry 2008). Recent research has identified propagule pressure as a general determinant of invasion success (Lockwood et al. 2005; Colautti et al. 2006; Hayes and Barry 2008; Simberloff 2009). Propagule pressure is a composite measure of the number of individuals in each introduction event (propagule size) and the number of separate introductions (propagule number), with the basic premise that as more individuals arrive in a recipient area, the likelihood of successful establishment increases (Lockwood et al. 2005; Simberloff 2009).

Despite the apparent importance of the number of individuals arriving in a new location on the resultant invasion success, the initial stages of the introduction process have generally been neglected (Floerl and Inglis 2005; Puth and Post 2005). Only individuals from a subset of species are actually transported (Floerl and Inglis 2005; Colautti et al. 2006). For those that are, the transportation phase (which is comprised of three steps: uptake, transit, disembarkation) is critically important as success is lowest at this stage, and it is where most unintentional introductions fail (Puth and Post 2005; Colautti et al. 2006; Ward et al. 2006; Tingley et al. 2010). Most attempts to identify the determinants of invasion success have focused on intentionally introduced species (i.e., plants, birds, mammals; Kraus 2003; Mack 2003) that, in effect, bypass this critical stage (Suarez et al. 2005; Hayes and Barry 2008). Deliberate introductions represent a biased subset of species, selected by humans due to specific traits, that have not been through the transportation filter (Suarez et al. 2005; Alpert 2006; Hayes and Barry 2008; Tingley et al. 2010). Thus, we require improved knowledge of the factors that influence the propensity for species to be inadvertently transported to new regions via human-mediated dispersal (Suarez et al. 2001; Wilson et al. 2009).

Behavior mediates how animals interact with their environment and should therefore play a pivotal role in their ability to transition through stages within the introduction process. Animals that reside or shelter within valuable commodities (e.g., fresh produce, timber, soil, plant material) have an enhanced likelihood of being inadvertently ensnared in freight or cargo (Gill et al. 2001; Ward et al. 2006; King et al. 2009; Toy and Newfield 2010). Species that occur in high densities in human-occupied environments will have distributions that overlap with major transport hubs and an increased opportunity for uptake into transport vectors (Floerl and Inglis 2005; Suarez et al. 2005; Toy and Newfield 2010). Animals living in urban environments are often more bold and exploratory (Short and Petren 2008; Evans et al. 2010), and these behaviors may also result in individuals actively searching and finding their way into freight, cargo, or personal effects (Sih et al. 2004a; Wilson et al. 2009). The frequent transportation of individuals, especially in groups, may increase the propagule pressure for the species; however, this will only be true for instances where the individuals survive transit and arrive in good condition (Simberloff 2009). The transport vector (e.g., truck, plane, ship) will strongly influence the length of transit and the conditions to which the stowaways are exposed (e.g., availability of food and water, oxygen levels, temperatures; Ruiz and Carlton 2003). Species that have a dormant life-cycle stage or a propensity to actively seek shelter may be better equipped for enduring the suboptimal or extreme temperatures experienced during transit (Colautti et al. 2006). Biosecurity checks may detect less than half of all hitchhikers, therefore species that actively hide in structurally complex cargo are more likely to evade detection during transit (Gill et al. 2001; Ward et al. 2006; Toy and Newfield 2010).

Upon arrival at the destination, individuals need to disembark from the transport vector, explore the new environment and seek out food, warmth, and suitable habitats (Colautti et al. 2006). Empirical studies demonstrate that transportation generally involves single individuals or small groups (Mack et al. 2000; Gill et al. 2001; Ward et al. 2006), which usually arrive at different times and often from multiple areas of the native range (Kolbe et al. 2004; Simberloff 2009). An implicit assumption, which has rarely been examined, is that individuals from these temporally or spatially separated propagules will be able to locate, recognize, and interact with each other in the introduced region (Lockwood et al. 2005; Colautti et al. 2006; Simberloff 2009). Small population size in these incipient introductions may lead to demographic or environmental stochasticity, or Allee effects and results in reduced effective population sizes and lower establishment success (Taylor and Hastings 2005; Blackburn et al. 2009; Tobin et al. 2011). Boldness and exploratory behavior may drive the subsequent spread of the established population across the introduced landscape (Rehage and Sih 2004; Russell et al. 2010), with traits such as aggression and behavioral flexibility often involved in outcompeting and displacing native species (Holway and Suarez 1999; Sol et al. 2002; 2008;). Subsequent human-assisted dispersal (“jump dispersal”) may enable some established species to rapidly spread across the introduced region (Suarez et al. 2001; Aubry et al. 2006; Wilson et al. 2009).

Since the factors that enable success at each introduction stage differ (Kolar and Lodge 2001; Colautti et al. 2006), certain behaviors may have a complementary influence across multiple phases, but counteractive impacts may also occur between stages. For instance, exploratory behavior may enhance the likelihood of uptake into transport vectors and subsequent establishment and spread, but might also increase the risk of detection during transit. Similarly, it has been demonstrated that invasive mosquitofish (*Gambusia affinis*) that disperse further are also more likely to be asocial (Cote et al. 2010), which could enhance their susceptibility to Allee effects. Here, we adopt a comparative approach (i.e., van Kleunen et al. 2010) to investigate whether pre-existing behavioral differences between two sympatric lizard species can explain their divergent invasion success during the introduction process.

**Figure 1:**
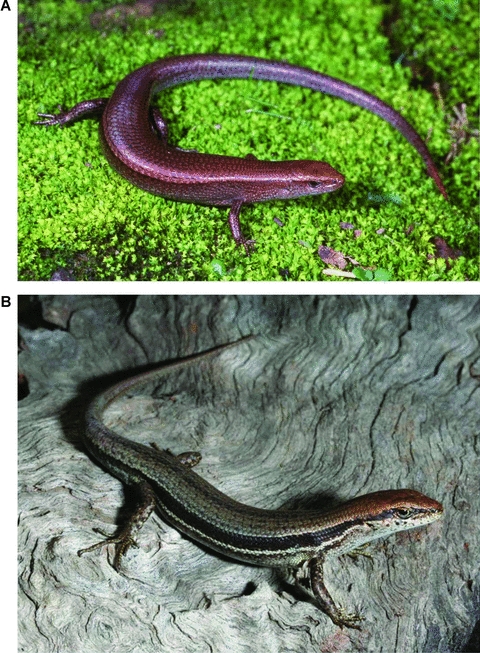
The two study species: (A) delicate skink (*L. delicata*), and (B) garden skink (*L. guichenoti*). Photographs: Nick Clemann.

The delicate skink (*Lampropholis delicata*) is the only Australian lizard species that has successfully established, and subsequently become invasive overseas (Lever 2003; Kraus 2009). It is one of the most abundant and widespread vertebrate species in eastern Australia, occurring across 26° of latitude from north Queensland to southern Tasmania (Wilson and Swan 2010; Chapple et al. 2011a). Throughout the majority of its native range (southeastern Queensland to southern Victoria), the delicate skink occurs in sympatry with the congeneric garden skink (*L. guichenoti*) (Wilson and Swan 2010; Chapple et al. 2011a,b). The two species are near identical in body size (∼35–55 mm adult snout-vent length [SVL]) and life history (e.g., oviparous, clutch size, reproductive ecology), and both species thrive in suburban habitats throughout southeastern Australia (Joss and Minard 1985; Prosser et al. 2006; Wilson and Swan 2010; Fig. 1; Table 1). However, while the delicate skink is an invasive species in the Hawaiian Islands, New Zealand, and Lord Howe Island (where it is called the rainbow or plague skink), the garden skink has never successfully established outside of Australia (Lever 2003; Kraus 2009). The two species do not appear to differ in their relative opportunity for transportation, as they both occur together in each of the transport hubs that have been identified, using molecular markers, as source regions for delicate skink introductions (Chapple et al., unpublished data). Both species have been intercepted in freight, cargo, and personal effects entering New Zealand (Gill et al. 2001; Kraus 2009; D. Chapple, unpublished data from MAF Biosecurity New Zealand interception records).

**Table 1:** Comparison of the morphology, life history, and ecology of the delicate skink (*L. delicata*) and the garden skink (*L. guichenoti*). The information was sourced from: Clarke (1965), Joss and Minard (1985), Simbotwe (1985), Greer (1989), Lunney et al. (1989), Kutt (1993), Wilson and Swan (2010)

Trait/characteristic	Delicate skink *L. delicata*	Garden skink *L. guichenoti*
Adult SVL (range)	35–55 mm	35–55 mm
Age at maturity	1 year	1 year
Lifespan	2–4 years	2–4 years
Reproductive mode	Oviparous	Oviparous
Clutch size (mean)	1–6 (∼3)	1–5 (∼3)
Communal nesting	Yes	Yes
Activity	Diurnal heliotherm	Diurnal heliotherm
Habitat preferences	Leaf litter and ground debris in rainforest, wet/dry sclerophyll forest, woodlands, heaths	Leaf litter and ground debris in wet/dry sclerophyll forest, woodlands, heaths
Microhabitat preferences	Sheltered	Open
Abundant in urban environments?	Yes	Yes
Latitudinal range	26° (16–42 °S)	12° (26–38 °S)
Diet	Invertebrate generalist	Invertebrate generalist

To investigate whether pre-existing behavioral differences might explain the differences in apparent propagule pressure, and subsequent establishment success, between these two *Lampropholis* skinks, we examined the grouping tendencies and exploratory behavior of delicate and garden skinks. Based on our own field observations, we predict that the two species will not differ in their grouping tendencies. However, given the repeated success of the delicate skink across multiple stages of the introduction process, we anticipate that it will be more exploratory across a variety of contexts relative to the garden skink. In addition, since the delicate skink prefers more sheltered microhabitats than the garden skink (Kutt 1993; Wilson and Swan 2010), we predict that it will also exhibit a greater propensity to seek shelter in novel environments, which may assist its survival during transit within transport vectors.

## Methods

### Collection and housing

The two *Lampropholis* species (*L. delicata* and *L. guichenoti*) were collected from suburban Sydney (33°53′39″ S, 151°10′44′′ E) in October 2009. This area represents one of the known source regions for successful delicate skink introductions (Chapple et al., unpublished data). Both species were abundant at the site, and were frequently observed to bask together. Upon collection, we determined the sex of the lizards (via the eversion of hemipenes in males), and took measurements of SVL and tail length (± 0.5 mm). Since, both gravidity (females of both species were gravid during the study period; Joss and Minard 1985) and tail loss are known to influence behavior in *Lampropholis* skinks (Downes and Shine 2001; Shine 2003), forty adult males (i.e., SVL > 35 mm), with full length tails (tail length > SVL), of each species were retained and transported to Monash University for laboratory experiments.

Lizards were housed in clear plastic containers (42 cm length × 31 cm width × 23 cm height) in a constant temperature room (20 ± 1°C) with a 14 L:10 D photoperiod (0600–2000 hours). The housing containers were lined with newspaper and each included a plastic shelter site and two terracotta basking tiles positioned under a heat lamp. This created a thermal gradient (20–35°C) within each container and enabled the skinks to thermoregulate freely. The lizards were fed three times weekly with crickets (*Acheta domesticus*) dusted with reptile supplement (Reptivite™) and provided with water ad libitum. Since *Lampropholis* skinks modify their behaviors following large meals (Shine 2003), we ensured that lizards were not fed in the 24 hours prior to each behavioral trial.

### Grouping tendencies

We conducted a series of dichotomous choice experiments to investigate the grouping behavior of the two *Lampropholis* species. The grouping tendencies of focal individuals from each species were examined in response to six different choice combinations: (1) conspecific group versus no lizards, (2) heterospecific group versus no lizards, (3) mixed species group versus no lizards, (4) conspecific group versus heterospecific group, (5) conspecific group versus mixed species group, and (6) heterospecific group versus mixed species group. These experiments enabled us to investigate whether the two species prefer to join a social grouping rather than remaining solitary, and if group composition (i.e., conspecific, heterospecific, or mixed species) influences their social behavior.

Individuals were randomly assigned as either focal or stimulus lizards. Once assigned, the lizards retained this designation throughout the experimental trials. To avoid potential order effects, each focal lizard was randomly allocated to a different sequence of experimental trials. The stimulus groups comprised four individuals (the mixed species groups comprised two individuals from each species), although the composition of individuals within these groups was changed between trials to ensure that the focal lizards did not repeatedly experience the same combination of stimulus lizards.

**Figure 2:**
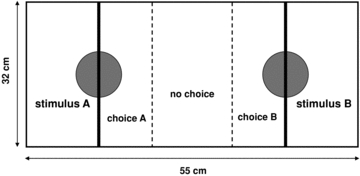
Overhead schematic view of the experimental setup for the dichotomous choice trials to investigate the grouping tendencies of *L. delicata* and *L. guichenoti*. A 40-W basking lamp was positioned above each basking site.

The trials were performed in large, opaque-walled test arenas (55 cm length × 32 cm width, 24-cm height). A black marker pen was used to designate five 11-cm segments along the length of the test arena (Fig. 2). A basking site, positioned under a 40-W heating lamp, was placed at each end of the arena, on the inner edge of the peripheral sections (Fig. 2). Each basking site was divided in half by a clear Perspex™ partition (10-cm high), which ran the width of arena (Fig. 2). The stimulus lizards were placed in these peripheral sections during the trials, which enabled the focal lizard to see, but not physically interact with them. This created three inner segments, with the two adjoining the basking sites designated as the “choice” zones for the respective stimuli, and the central one considered to be a “no choice” or neutral region (Fig. 2).

The focal lizard was placed in the neutral region under an open topped, clear plastic container 10 min prior to the commencement of the trial to enable it to acclimatize to the test arena. At the start of the trial, the plastic container was removed and the lizard was able to freely move and select one of the two basking sites. The temperature underneath the heat lamps (∼35°C) was substantially higher than the ambient temperature (20°C), prompting the lizards to use the basking sites. Each trial lasted for 45 min and was video recorded (Signet 4 channel digital video recorder, Signet Electronic Systems Inc., Norwell, MA, USA) using a camera suspended over each test arena. Between each trial, the test arena and equipment was wiped with 70% ethanol and washed with hot water and detergent to remove any chemical cues from the trial. The trials were subsequently played back using PCviewer v 2.3.8 surveillance software and the time spent associating with each stimulus was recorded using Etholog v2.2.5 (Ottoni 2000). The data were arcsine square root transformed, and then analyzed using paired *t*-tests (Quinn and Keough 2002) in R v2.9.1 (R Development Core Team 2009). Significance levels were adjusted according to the Bonferroni correction procedure (Rice 1989) for multiple comparisons as described by Holm (1979).

### Exploratory behavior

The activity in novel environments, tendency to seek shelter, and exploratory behavior across three different contexts was compared between the two *Lampropholis* species.

1. *Activity in novel environments and tendency to seek shelter*. The basal nondirected activity of the two lizard species was examined in an open opaque-walled test arena (55 cm length × 32 cm width, 24-cm height), with twenty grid squares (8 × 11 cm) marked on the floor. All trials were conducted at 25°C. The lizard was placed under a clear plastic holding container for 10 min prior to the commencement of the trial to acclimate to the test arena. At the start of the trial, the plastic container was removed and the lizard was able to freely move around the test arena for 45 min. Each trial was video recorded and during playback the activity of each lizard (taken as the number of transitions between grid squares) and the time spent in the 14 peripheral grid squares (vs. six interior ones) recorded. A second trial was conducted as per the first trial, except that a shelter site (two small upturned terracotta dishes) was placed in one of the internal grid squares. This trial aimed to compare the tendency of both species to hide or seek shelter in a novel environment. Each trial was video recorded and the activity, time spent in the peripheral regions, and time spent in the shelter recorded. The time spent in shelter and around the perimeter was arcsine square root transformed prior to analysis. The activity, time spent in the shelter, and periphery of the arena in each species was compared using independent *t*-tests.
2. *Locating new environments and resources*. We compared the exploratory behavior of the two *Lampropholis* species in three different contexts: (a) ability to find novel environments, (b) capacity to traverse obstacles to find novel environments, and (c) aptitude for seeking out an essential resource (i.e., basking site) in a structurally complex novel environment. Each of these trials was conducted using modifications of the test arena (55 cm length × 32 cm width, 24-cm height). Trials were carried out at an ambient temperature of 25°C, unless stated otherwise.
  In the first trial, a black opaque Perspex partition was placed 20 cm from the end of the test arena. The only way that lizards were able to move from the starting chamber (35 cm length × 32 cm width) to the area on the other side of a partition was through a PVC (Polyvinyl chloride) tube (4-cm diameter, 10 cm in length), located 2.5 cm above ground level (i.e., the lizards were not able to see through to the other chamber). In the second trial, a similar experimental setup was used, except that the movement between two areas involved overcoming an obstacle rather than moving through a PVC pipe. The Perspex partition was a trapezium shape (flush with the edge of arena at the base, but with a 1.5 cm gap at 10-cm height). This required the lizards to climb and squeeze through a small gap (designed to replicate getting into freight or cargo) to reach the other chamber. The final trial involved traversing a structurally complex environment to access an elevated basking site (∼35°C, compared to an ambient temperature of 21°C). During the introduction process, it is essential for lizards to be able to locate thermally suitable environments to maintain their body temperature. The lizards were only able to access the basking site by walking up a textured wooden walkway (width 3 cm), placed at a 30° angle, to the elevated (10-cm height) basking site.
  In each trial, the lizards were given 10 min to acclimate to the test arena. During the trial, the time taken to reach the new chamber or reach the basking site was recorded. Skinks that did not reach the goal during the 45-min trials were “right censored” (i.e., assigned the full trial time; Klein and Moeschlberger 2003). Nonparametric survival analysis, taken as the time until an event, was used to compare the exploratory behavior of the two *Lampropholis* species.

## Results

Neither the delicate skink nor garden skink exhibited a preference for basking alone or as part of a group, a result that was consistent regardless of the group composition (Table 2). Individuals of both species associated freely with groups comprising conspecifics, heterospecifics, and a mixture of both. Although the garden skink displayed a slight preference for basking with mixed species groupings, this was not statistically significant following Bonferroni correction (Table 2).

**Table 2:** Grouping tendencies of *L. delicata* and *L. guichenoti*, indicating the proportion of time spent associating with each stimulus during the six dichotomous choice experiments. The results of the paired *t*-tests are indicated. No comparisons were found to be statistically significant following Bonferroni correction

				*t*-test		
				
Experiment	Species	Stimulus A	Stimulus B	*t*	df	*P*
		Conspecific	No lizard			
Conspecific versus no lizard	*delicata*	0.36	0.64	–1.72	17	0.10
	*guichenoti*	0.59	0.41	0.82	18	0.42
		Heterospecific	No lizard			
Heterospecific versus no lizard	*delicata*	0.63	0.37	–1.79	17	0.09
	*guichenoti*	0.34	0.66	1.64	17	0.12
		Mixed species	No lizard			
Mixed species versus no lizard	*delicata*	0.42	0.58	–1.72	17	0.22
	*guichenoti*	0.58	0.42	0.82	18	0.40
		Conspecific	Heterospecific			
Conspecific versus heterospecific	*delicata*	0.50	0.50	–0.09	17	0.93
	*guichenoti*	0.52	0.48	0.40	19	0.69
		Conspecific	Mixed species			
Conspecific versus mixed species	*delicata*	0.53	0.47	0.41	17	0.69
	*guichenoti*	0.42	0.58	–0.88	17	0.39
		Heterospecific	Mixed species			
Heterospecfic versus mixed species	*delicata*	0.46	0.54	–0.67	17	0.51
	*guichenoti*	0.33	0.67	–2.15	16	0.05

**Figure 3:**
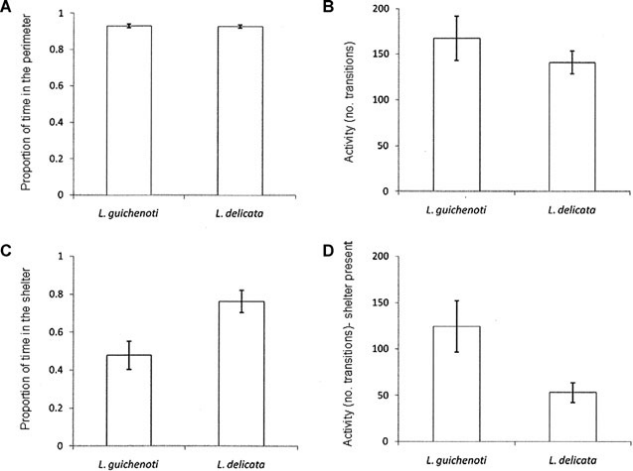
Comparison of the two *Lampropholis* species in their (A) time spent in the perimeter of an open novel environment, (B) activity in an open environment, (C) time spent using the shelter site, and (D) activity when a shelter site was available.

When placed into a novel open environment, both species exhibited a similar level of nondirected activity (*t* = –0.96, df = 26.80, *P* = 0.34; Fig. 3B) and time spent in the perimeter of the test arena (*t* = 0.04, df = 32.16, *P* = 0.34; Fig. 3A). However, when provided with an opportunity to seek shelter the delicate skink spent more time hiding (*t* = 3.13, df = 34.23, *P* = 0.02; Fig. 3C) and was less active (*t* = –2.41, df = 23.04, *P* = 0.02; Fig. 3D) compared to the garden skink.

**Figure 4:**
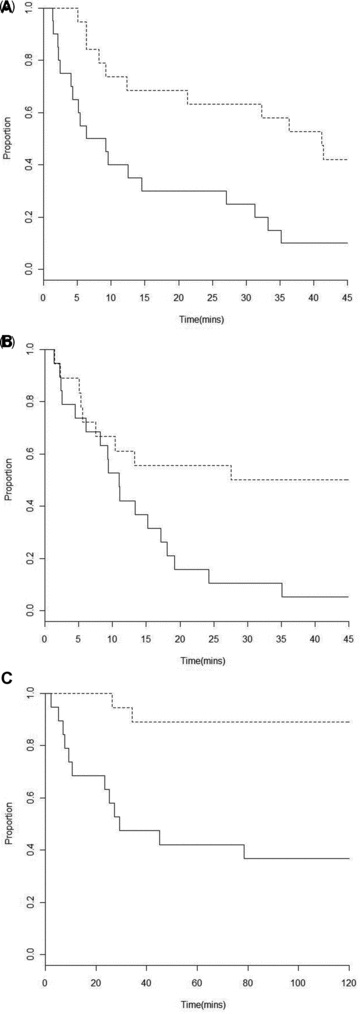
The time taken by *L. delicata* (solid line) and *L. guichenoti* (dashed line) to reach a new environment or a basking site resource. (A) Moving through a linking tunnel to a new area, (B) traversing an obstacle to reach a new area, and (C) reaching a raised basking site.

The delicate skink was substantially more exploratory than the garden skink, and was more likely to find the pathways through to the novel environments or resources (Fig. 4). In addition, the delicate skinks were quicker to reach these goals (Fig. 4). This result was consistent for moving through the tunnel to a new environment (*z* = 3.03, df = 39, *P* = 0.002; Fig. 4A), traversing an obstacle (*z* = 3.26, df = 37, *P* < 0.001; Fig. 4B), and reaching a raised basking site (*z* = 3.10, df = 37, *P* < 0.001; Fig. 4C).

## Discussion

Our study identified several pre-existing behavioral differences between the two sympatric *Lampropholis* species that might contribute to the success of the delicate skink across multiple stages of the introduction process. Although the nondirected activity was similar in both species, the delicate skink was more exploratory than the garden skink. This enabled the delicate skink to be more effective in finding new environments and essential resources (i.e., a basking site), and to do so more quickly. However, when provided with a shelter site, the delicate skink was more likely to decrease its activity and spend more time in the refuge. Such a tendency to actively hide might act to decrease the detection of the delicate skink during their ensnarement in freight and cargo. We acknowledge that there are potential limitations of two-species comparative studies (i.e., Garland and Adolph 1994); however, our study was restricted to focusing on *L. delicata* and *L. guichenoti* as these are the only two *Lampropholis* species that have distributions that encompass the known source regions for the invasive populations (i.e., equal “opportunity” for transportation). Nonetheless, we believe that the current study provides valuable insights into the behavioral traits that may enhance transition success across various stages of the introduction process.

While success at each phase of the introduction process is highly variable (Williamson and Fitter 1996; Jeschke and Strayer 2005), most stowaways fail to persist throughout transportation and initial establishment in the new area (Kolar and Lodge 2001). These initial stages of the introduction process have generally been understudied (Floerl and Inglis 2005; Puth and Post 2005); however, they appear to be key to understanding why the two *Lampropholis* species exhibit divergent invasion success. Despite being transported regularly in freight and cargo, the garden skink has repeatedly failed to establish in areas outside of its native range (Gill et al. 2001; Kraus 2009). In contrast, the delicate skink has repeatedly been a successful invader, with established populations in the Hawaiian Islands, New Zealand, and Lord Howe Island (Lever 2003; Kraus 2009). Molecular data indicate that it has established in one of these locations on multiple independent occasions (Chapple et al., unpublished data). We therefore examine how the behavioral differences between the two species might enable the delicate skink to be more successful during uptake into transport vectors, surviving transit, and the initial establishment in nonnative regions.

### The delicate skink has a greater propensity for successful human-assisted dispersal

The delicate skink was shown to be substantially more exploratory than the garden skink, even traversing obstacles to locate new environments. The introduction process acts as a sequential selective filter, with only a subset of individuals successfully transitioning through a particular stage (Sih et al. 2004a, b). Exploratory delicate skink individuals might be more likely to locate and get into freight, cargo, and personal effects, and be transported to areas outside of their native range. This result is consistent with an analysis of the biosecurity records for lizards entering New Zealand, a country where the delicate skink is the only introduced lizard species to successfully establish (Gill et al. 2001). These records reveal that ∼75% of interceptions involve individual lizards (Gill et al. 2001). While previous studies have indicated a link between exploratory behavior and the postestablishment dispersal and spread of an invasive species (Rehage and Sih 2004; Cote et al. 2010; 2011; Fogarty et al. 2011), its role in the initial inadvertent transportation of stowaways has rarely been emphasized (Holway and Suarez 1999).

The potential importance of exploratory behavior in unintentional human-mediated transportation may be reflected in the relatively low incidence of the garden skink as a hitchhiker. While the garden skink thrives in human-modified urban environments (Prosser et al. 2006), and occurs sympatrically with the delicate skink in all the major transport hubs (Wilson and Swan 2010), it is not transported as frequently (Gill et al. 2001; Kraus 2009; D. Chapple, unpublished data from MAF Biosecurity New Zealand interception records). Our study suggests that this might be due to the garden skink not being as exploratory as the delicate skink. However, successful transportation also requires that stowaways remain within the commodity or cargo prior to and during transit. Our results indicate that garden skinks might be less likely to seek shelter within freight and cargo after locating the items and, thus, become ensnared within transported materials less often. The transportation of garden skinks might therefore have a greater reliance on passive uptake of the commodities in which the individuals are residing. Both *Lampropholis* species are known to bask and overwinter together within fallen logs, under rocks, and within plant material (Greer 1989, this study). These materials represent commodities that comprise a large component of freight and cargo consignments (Gill et al. 2001; Toy and Newfield 2010), and even nonexploratory *Lampropholis* individuals might be transported in this manner (Lever 2003; Hutchinson et al. 2005).

Despite being more exploratory than the garden skink, the delicate skink displayed a stronger tendency to hide when provided with a shelter site. This may stem from the delicate skink preferring more closed microhabitats compared to the garden skink (Kutt 1993) However, individuals that actively hide within freight and cargo are less likely to be detected during transit and biosecurity border checks (Colautti et al. 2006; Ward et al. 2006; Toy and Newfield 2010). Only those individuals that avoid detection and arrive in good health have an opportunity to establish in the new environment in which they are deposited (Colautti et al. 2006; Simberloff 2009). The delicate skink therefore appears to exhibit two complementary behavioral traits (exploratory behavior, tendency to hide) that enhances its propensity for successful transportation. Seeking shelter may also enhance survival during transit, particularly within transport vectors (e.g., planes, cargo ships, trucks) that may be exposed to temperature extremes (Colautti et al. 2006). Refuge sites within the cargo materials may buffer the stowaways from these extremes and enable them to survive transit. Indeed, ∼90% of delicate skinks intercepted entering New Zealand were alive when detected (Gill et al. 2001; D. Chapple, unpubl. data from MAF Biosecurity New Zealand interception records), and therefore most transportation events are likely to result in viable propagules being deposited in the recipient area.

### Why has the delicate skink been more successful at establishing in new locations?

Surviving transportation is only the first stage of the introduction process, and individuals need to disembark from the transport vector. Since exiting the cargo may be considered the logical opposite of the initial ensnarement within it, exploratory behavior is also likely to have a central role in establishment. The delicate skink exhibited a greater tendency to explore novel environments and was more adept at locating and utilizing basking sites. Exploratory behavior is important for colonizing individuals as it enables them to familiarize themselves with the new environment and locate essential resources (Sih et al. 2004a,b; Russell et al. 2010). For instance, Iberian wall lizards (*Podarcis dispanica*) that were more exploratory also habituated to new environments and predators more readily than other individuals (Rodriguez–Prieto et al. 2011). However, exploratory individuals are often better dispersers that exhibit lower social tendencies (Rehage and Sih 2004; Cote et al. 2010), which may decrease the density of the introduced populations and lead to Allee effects (Taylor and Hastings 2005; Tobin et al. 2011).

Both *Lampropholis* species are potentially susceptible to Allee effects since they are communal nesters (Greer 1989) and group size influences antipredator vigilance behavior (Downes and Hoefer 2004). However, the grouping tendencies of the two species were similar with no strong preference evident for individuals basking alone or in groups. Interestingly, the group composition did not influence the social tendencies of the two species with individuals not actively discriminating among conspecific, heterospecific, and mixed species aggregations. This may enable individuals from temporally separated propagules to locate and interact with each other, even those originating from different regions of the species native range. Although we only examined males in the current study, if this result holds true for groups comprising both males and females, it may enhance the likelihood of admixture (Kolbe et al. 2004; Simberloff 2009) occurring within the introduced range of *Lampropholis* species. Indeed, molecular evidence indicates that admixture is present in at least one invasive population of the delicate skink (Chapple et al., unpublished data).

Although exploratory behavior in the delicate skink might also be associated with greater dispersal tendencies (e.g., Rehage and Sih 2004; Cote et al. 2010), there is anecdotal evidence in New Zealand and the Hawaiian Islands that it is predominantly spread via human-mediated jump dispersal (Baker 1979; Chapple et al., unpublished data). The high propensity to get transported in freight and cargo has enabled the delicate skink to spread rapidly throughout these two archipelagos, including across water barriers. Similarly, high rates of human-mediated dispersal has been documented in the introduced range of invasive ants (*Linepithema humile, Solenopsis invicta*; Suarez et al. 2001; King et al. 2009), land snails (*Xeropicta derbentina*; Aubry et al. 2006), and the cane toad (*Bufo marinus*; White and Shine 2009), enabling more rapid spread than via natural dispersal alone (Wilson et al. 2009). Thus, behavioral traits associated with human-assisted dispersal may contribute to success across multiple stages of the introduction process.

## Conclusions

While the two *Lampropholis* species might have a similar “opportunity” for human-assisted transportation, the delicate skink appears to have several pre-existing behavioral traits that contribute to its successful transition through the introduction process. Although the divergent invasion success of the two species might be due to differences in propagule pressure, our study has illustrated how behavioral mechanisms may underlie both propagule size and number. While clear differences in exploratory behavior were found between the two species, not all delicate skink individuals successfully located the new environments and basking site. This indicates that there is substantial individual variation in these behaviors within the species and may result in only a select subset of individuals progressing through each introduction stage. The repeated invasion success of the delicate skink provides an ideal system in which to conduct future investigation into the behavioral traits that underlie the success of species across multiple introduction stages.

## References

[b1] Alpert P (2006). The advantages and disadvantages of being introduced. Biol. Invas.

[b2] Aubry S, Labaune C, Magnin F, Roche P, Kiss L (2006). Active and passive dispersal of an invading land snail in Mediterranean France. J. Anim. Ecol.

[b3] Baker JK (1979). The rainbow skink, *Lampropholis delicata*, in Hawaii. Pacific Sci.

[b4] Blackburn TM, Cassey P, Lockwood JL (2009). The role of species traits in the establishment success of exotic birds. Glob. Change Biol.

[b5] Chapple DG, Hoskin CJ, Chapple SNJ, Thompson MB (2011a). Phylogeographic divergence in the widespread delicate skink (*Lampropholis delicata*) corresponds to dry habitat barriers in eastern Australia. BMC Evol. Biol.

[b6] Chapple DG, Chapple SNJ, Thompson MB (2011b). Biogeographic barriers in south-eastern Australia drive phylogeographic divergence in the garden skink, *Lampropholis guichenoti*. J. Biogeogr.

[b7] Clarke CJ (1965). A comparison between some Australian five-fingered lizards of the genus *Leiolopisma* Dumeril & Bibron (Lacertilia: Scincidae). Aust. J. Zool.

[b8] Colautti RI, Girgorovich IA, MacIsaac HJ (2006). Propagule pressure: a null model for biological invasions. Biol. Invas.

[b9] Cote J, Fogarty S, Brodin T, Weinersmith K, Sih A (2011). Personality-dependent dispersal in the invasive mosquitofish: group composition matters. Proc. R. Soc. Lond. B.

[b10] Cote J, Fogarty S, Weinersmith K, Brodin T, Sih A (2010). Personality traits and dispersal tendency in the invasive mosquitofish (*Gambusia affinis*). Proc. R. Soc. Lond. B.

[b11] Downes S, Hoefer AM (2004). Vigilance in lizards: interactions between group size and predation risk. Anim. Behav.

[b12] Downes S, Shine R (2001). Why does tail loss increase a lizard's later chances of being consumed by snake predators?. Ecology.

[b13] Evans J, Boudreau K, Hyman J (2010). Behavioural syndromes in urban and rural populations of song sparrows. Ethology.

[b14] Floerl O, Inglis GJ (2005). Starting the invasion pathway: the interaction between source populations and human transport vectors. Biol. Invas.

[b15] Fogarty S, Cote J, Sih A (2011). Social personality polymorphism and the spread of invasive species: a model. Am. Nat.

[b16] Garland T, Adolph SC (1994). Why not to do two-species comparative studies: limitations on inferring adaptation. Physiol. Zool.

[b17] Gill BJ, Bejakovich D, Whitaker AH (2001). Records of foreign reptiles and amphibians accidentally imported to New Zealand. New Zeal. J. Zool.

[b18] Greer AE (1989). The biology and evolution of Australian lizards.

[b19] Hayes KR, Barry SC (2008). Are there any consistent predictors of invasion success?. Biol. Invas.

[b20] Holm S (1979). A simple sequentially rejective multiple test procedure. Scand. J. Stat.

[b21] Holway DA, Suarez AV (1999). Animal behavior: an essential component of invasion biology. Trends Ecol. Evol.

[b22] Hutchinson MN, Thompson MB, Stewart JR (2005). *Lampropholis delicata* (Delicate skink, Rainbow skink). Introduction. Herpetol. Rev.

[b23] Jeschke JM, Strayer DL (2005). Invasion success of vertebrates in Europe and North America. Proc. Nat. Acad. Sci. U.S.A.

[b24] Joss JMP, Minard JA (1985). On the reproductive cycles of *Lampropholis guichenoti* and *L. delicata* (Squamata: Scincidae) in the Sydney region. Aust. J. Zool.

[b25] King JR, Tschinkel WR, Ross KG (2009). A case study of human exacerbation of the invasive species problem: transport and establishment of polygene fire ants in Tallahassee, Florida, USA. Biol. Invas.

[b26] Klein J, Moeschlberger M (2003). Statistics for biology and health: survival analysis, techniques for censored and truncated data.

[b27] Kolar CS, Lodge DM (2001). Progress in invasion biology: predicting invaders. Trends Ecol. Evol.

[b28] Kolbe JJ, Glor RE, Schettino LR, Lara AC, Larson A, Losos JB (2004). Genetic variation increases during biological invasion by a Cuban lizard. Nature.

[b29] Kraus F, Ruiz GM, Carlton JT (2003). Invasion pathways for terrestrial vertebrates. Invasive species: vectors and management strategies.

[b30] Kraus F (2009). Alien reptiles and amphibians: a scientific compendium and analysis.

[b31] Kutt A, Lunney D, Ayers D (1993). Initial observations on the effect of thinning eucalypt regrowth on heliothermic skinks in lowland forest, East Gippsland, Victoria. Herpetology in Australia- a diverse discipline.

[b32] Lever C (2003). Naturalized reptiles and amphibians of the world.

[b33] Lockwood JL, Cassey P, Blackburn T (2005). The role of propagule pressure in explaining species invasions. Trends Ecol. Evol.

[b34] Lockwood JL, Hoopes MF, Marchetti MP (2007). Invasion ecology.

[b35] Lunney D, Ashby E, Grigg J, O'Connell M (1989). Diets of scincid lizards *Lampropholis guichenoti* (Dumeril & Bibron) and *L. delicata* (De Vis) in Mumbulla State Forest on the south coast of New South Wales. Aust. Wildl. Res.

[b36] Mack RN, Ruiz GM, Carlton JT (2003). Global plant dispersal, naturalization, and invasion: pathways, modes and circumstances. Invasive species: vectors and management strategies.

[b37] Mack RN, Simberloff D, Lonsdale WM, Evans H, Clout M, Bazzaz FA (2000). Biotic invasions: causes, epidemiology, global consequences, and control. Ecol. Appl.

[b38] Ottoni E (2000). Etholog 2.2: a tool for the transcription and timing of behavioural observation sessions. Behav. Res. Meth.

[b39] Prosser C, Hudson S, Thompson MB (2006). Effects of urbanization on the behavior, performance and morphology of the Garden skink, *Lampropholis guichenoti*. J. Herpetol.

[b40] Puth LM, Post DM (2005). Studying invasion: have we missed the boat?. Ecol. Lett.

[b41] Quinn GP, Keough MJ (2002). Experimental design and data analysis for biologists.

[b42] R Development Core Team (2009). The foundation for statistical computing.

[b43] Rehage JS, Sih A (2004). Dispersal behavior, boldness, and the link to invasiveness: a comparison of four *Gambusia* species. Biol. Invas.

[b44] Rice WR (1989). Analysing tables of statistical tests. Evolution.

[b45] Rodriguez-Prieto I, Martin J, Fernandez-Juricic E (2011). Individual variation in behavioural plasticity: direct and indirect effects of boldness, exploration and sociability on habituation to predators in lizards. Proc. R. Soc. Lond. B.

[b46] Ruiz GM, Carlton JT (2003). Invasive species. Vectors and management strategies.

[b47] Russell JC, McMorland AJC, MacKay JWB (2010). Exploratory behaviour of colonizing rats in novel environments. Anim. Behav.

[b48] Shine R (2003). Locomotor speeds of gravid lizards: placing ‘costs of reproduction’ within an ecological context. Funct. Ecol.

[b49] Short KH, Petren K (2008). Boldness underlies foraging success of invasive *Lepidodactylus lugubris* geckos in the human landscape. Anim. Behav.

[b50] Sih A, Bell AM, Johnson JC, Ziemba RE (2004a). Behavioral syndromes: an integrative overview. Quart. Rev. Biol.

[b51] Sih A, Bell A, Johnson JC (2004b). Behavioral syndromes: an ecological and evolutionary overview. Trends Ecol. Evol.

[b52] Simberloff D (2009). The role of propagule pressure in biological invasions. Ann. Rev. Ecol. Evol. Syst.

[b53] Simbotwe MP, Grigg G, Shine R, Ehmann H (1985). Sexual dimorphism and reproduction of *Lampropholis guichenoti* (Lacertilia: Scincidae). Biology of Australasian frogs and reptiles.

[b54] Sol D, Bacher S, Reader SM, Lefebvre L (2008). Brain size predicts the success of mammal species introduced to novel environments. Am. Nat.

[b55] Sol D, Timmermans S, Lefebvre L (2002). Behavioural flexibility and invasion success in birds. Anim. Behav.

[b56] Suarez AV, Holway DA, Case TJ (2001). Patterns of spread in biological invasions dominated by long-distance jump dispersal: insights from Argentine ants. Proc. Nat. Acad. Sci. U.S.A.

[b57] Suarez AV, Holway DA, Ward PS (2005). The role of opportunity in the unintentional introdution of nonnative ants. Proc. Nat. Acad. Sci. U.S.A.

[b58] Suarez AV, Tsutsui ND (2008). The evolutionary consequences of biological invasions. Mol. Ecol.

[b59] Taylor CM, Hastings A (2005). Allee effects in biological invasions. Ecol. Lett.

[b60] Tingley R, Romagosa CM, Kraus F, Bickford D, Phillips BL, Shine R (2010). The frog filter: amphibian introduction bias driven by taxonomy, body size and biogeography. Global Ecol. Biogeogr.

[b61] Tobin PC, Berec L, Liebhold AM (2011). Exploiting Allee effects for managing biological invasions. Ecol. Lett.

[b62] Toy SJ, Newfield MJ (2010). The accidental introduction of invasive animals as hitchhikers through inanimate pathways: a New Zealand perspective. Rev. Sci. Tech.

[b63] van Kleunen M, Dawson W, Schlaepfer D, Jeschke JM, Fischer M (2010). Are invaders different? A conceptual framework of comparative approaches for assessing determinants of invasiveness. Ecol. Lett.

[b64] Ward DF, Beggs JR, Clout MN, Harris RJ, O'Connor S (2006). The diversity and origin of exotic ants arriving in New Zealand via human-mediated dispersal. Divers. Distrib.

[b65] White AW, Shine R (2009). The extra-limital spread of an invasive species via ‘stowaway’ dispersal: toad to nowhere?. Anim. Conerv.

[b66] Williiamson M, Fitter A (1996). The varying success of invaders. Ecology.

[b67] Wilson JRU, Dormontt EE, Prentis PJ, Lowe AJ, Richardson DM (2009). Something in the way you move: dispersal pathways affect invasion success. Trends Ecol. Evol.

[b68] Wilson S, Swan G (2010). A complete guide to Reptiles of Australia.

